# Microglia-orchestrated neuroinflammation and synaptic remodeling: roles of pro-inflammatory cytokines and receptors in neurodegeneration

**DOI:** 10.3389/fncel.2025.1700692

**Published:** 2025-11-10

**Authors:** Guang Yang, Xiao Xu, Waimao Gao, Xingyu Wang, Yan Zhao, Ying Xu

**Affiliations:** 1 Department of Physiology, School of Integrative Medicine, Shanghai University of Traditional Chinese Medicine, Shanghai, China; 2 Shanghai Institute of Traditional Chinese Medicine for Mental Health, Shanghai, China

**Keywords:** microglia, neuroinflammation, cytokines/receptors, synaptic modification, synaptic plasticity, synaptic pruning, neurodegeneration

## Abstract

Microglia, the innate immune cells of the central nervous system (CNS), play essential roles in maintaining neural homeostasis through dynamic interactions with neurons and other brain structures. While their protective functions are well-established, recent studies have illuminated the detrimental consequences of sustained microglial activation in the context of neurodegeneration. In particular, overactivated microglia contribute to neuroinflammation and induce synaptic alterations through the release of pro-inflammatory cytokines and engagement of specific receptors. These interactions disrupt synaptic structure and function, compromising connectivity, plasticity, and cognitive processes. Notably, neuronal synapses are primary targets of such inflammation-driven dysfunction, where prolonged exposure to cytokines such as interleukin-1β (IL-1β), interleukin-6 (IL-6), and tumor necrosis factor-*α* (TNF-α), and signaling via receptor systems including cluster of differentiation-200 (CD200)/CD200 receptor (CD200R), C-X3-C motif chemokine ligand 1 (CX3CL1)/CX3C receptor 1 (CX3CR1), colony-stimulating factor 1 (CSF1)/CSF1 receptor (CSF1R), and interferon-*γ* (IFN-γ)/IFN-γ receptor (IFN-γR), lead to impaired learning, excitotoxicity, and neurodegenerative progression. This review synthesizes emerging evidence on the mechanisms by which microglia-mediated immune responses regulate synaptic remodeling, emphasizing the roles of pro-inflammatory cytokines and their receptors in neurodegenerative disorders.

## 1 Introduction

Microglia, the resident immune cells of the brain, originate from primitive c-kit(+) erythromyeloid precursors in the yolk sac and migrate into the brain parenchyma during early development. This population is self-sustaining under normal conditions, with peripheral macrophages contributing only in pathological states when the blood–brain barrier is compromised (Ginhoux et al., 2010). Beyond their well-established immunological functions in maintaining central nervous system (CNS) homeostasis, microglia have gained recognition for their critical involvement in synaptic modification, both during neural development and in the pathogenesis of neurodegenerative diseases.

**Figure 1:**
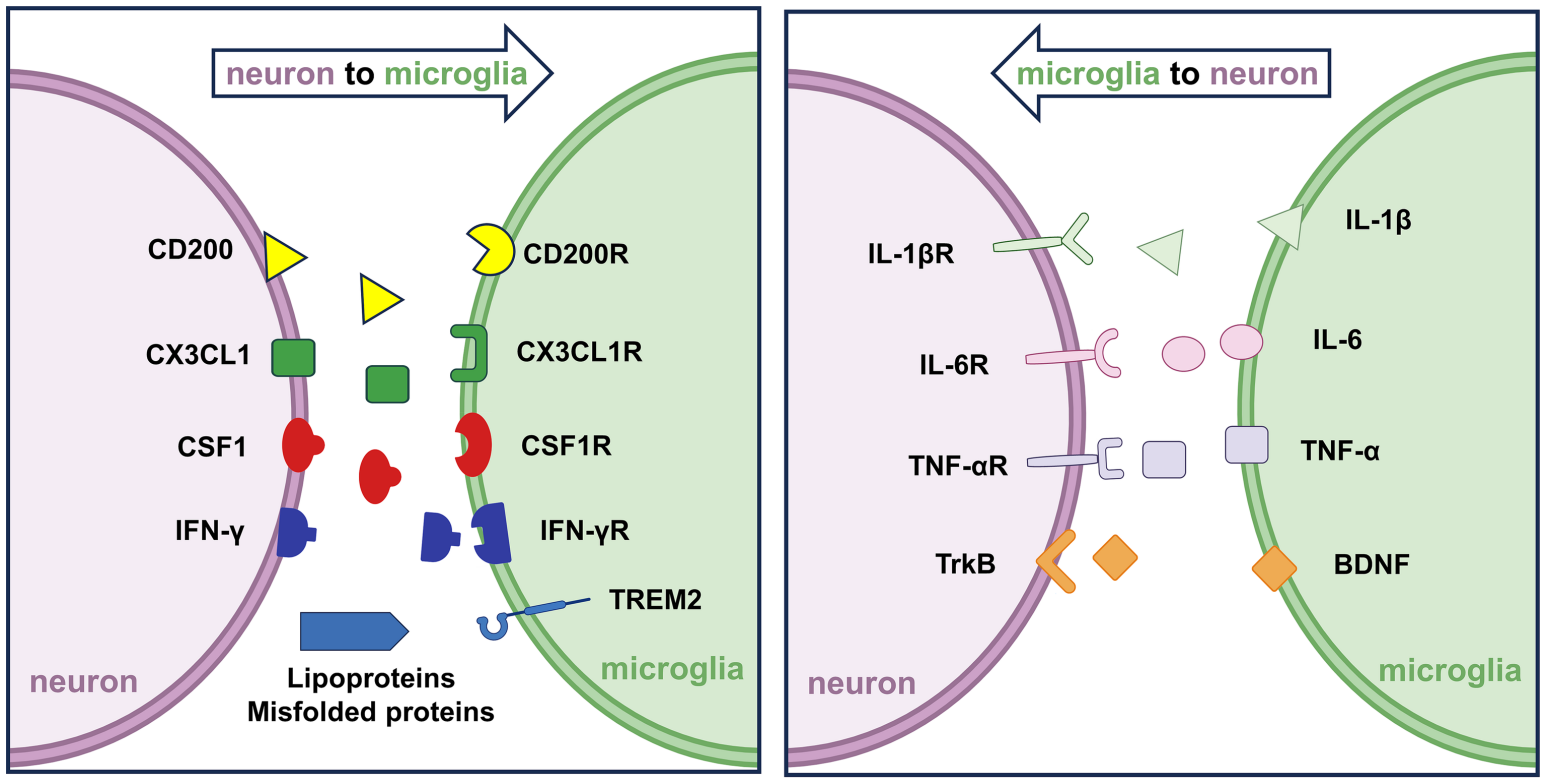
Neuron–microglia interactions under physiological conditions. **(Left)** Neurons secrete signaling molecules such as CX3CL1, CSF1, and IFN-γ, which bind to their respective microglial receptors, CX3CL1R, CSF1R, and IFN-γR, to regulate microglial surveillance and activation. Additionally, neuronal CD200 interacts with microglial CD200R to maintain microglial quiescence. **(Right)** Activated microglia release pro-inflammatory cytokines, including IL-1β, IL-6, and TNF-*α*, which bind to neuronal receptors IL-1βR, IL-6R, and TNF-αR, respectively. These bidirectional interactions coordinate immune homeostasis and contribute to the maintenance of neuronal and synaptic integrity.

Microglia typically display two phenotypic states: “resting” and “activated.” The term “resting” is somewhat misleading, as these cells are highly dynamic even under physiological conditions. Characterized by small somata and extensively branched processes, resting microglia continuously survey the CNS microenvironment and interact with surrounding neural elements, particularly neurons and synapses. Through their motile processes, they engage in synaptic remodeling, support CNS repair, and respond to immune challenges arising from peripheral insults (Figure 1). In pathological contexts, such as Alzheimer’s disease (AD) (Hopp et al., 2018), Parkinson’s disease (PD) (Rabaneda-Lombarte et al., 2021), amyotrophic lateral sclerosis (ALS) (Cox et al., 2022), multiple sclerosis (MS) (Cignarella et al., 2020), stroke (He et al., 2019), and chronic pain (Zhou et al., 2019), microglia undergo activation in response to injury or inflammatory stimuli. This transformation encompasses a series of biological processes, including local proliferation, morphological changes, migration, antigen presentation, phagocytosis, and the release of a wide array of signaling molecules (Kaur et al., 2019).

Activated microglia may exert either protective or deleterious effects, depending on the nature and duration of the stimuli. In the early stages of CNS injury, they clear debris, protein aggregates, and apoptotic cells, while releasing chemokines, cytokines, and neurotrophic factors that support immune regulation and tissue repair. However, persistent microglial activation can contribute to neuropathology. Chronically active microglia release pro-inflammatory cytokines and engage receptor-mediated signaling cascades that disrupt synaptic function and plasticity. Notably, elevated levels of interleukin-1β (IL-1β), interleukin-6 (IL-6), tumor necrosis factor-*α* (TNF-α), cluster of differentiation-200 (CD200)/CD200 receptor (CD200R), C-X3-C motif chemokine ligans 1 (CX3CL1)/CX3C receptor 1 (CX3CR1), colony-stimulating factor 1 (CSF1)/CSF1 receptor (CSF1R), and interferon-*γ* (IFN-γ)/IFN-γ receptor 1 (IFNGR1)-IFN-γ receptor 2 (IFNGR2) have been implicated in neuroinflammation (Rabaneda-Lombarte et al., 2021; Yang et al., 2023; van der Maten et al., 2017; Yang et al., 2018; Panagiotakopoulou et al., 2020). Such dysregulated signaling interferes with key processes like dendritic spine remodeling (Huang et al., 2021), synaptic phagocytosis (Wang et al., 2021), and neurotransmission (Yang et al., 2018), ultimately contributing to excitotoxicity and the progression of neurodegeneration.

Despite increasing research interest, the precise mechanisms through which microglial cytokines and receptors influence synaptic structures remain incompletely understood, and controversy persists regarding the dualistic role of microglia in disease. This review highlights recent advances in our understanding of overactive microglia and their contribution to neuroinflammation-driven synaptic dysfunction in neurodegenerative disorders. In particular, we focus on how pro-inflammatory cytokines and their receptors orchestrate microglia-mediated signaling networks that influence synaptic structure and function under pathological conditions. By synthesizing emerging mechanistic insights, this review aims to elucidate the complex interplay between microglial signaling and synaptic pathology in neurodegeneration.

## 2 Microglia-mediated immune responses regulate synaptic modification

As the principal immune cells of the CNS, microglia are fundamental to maintaining brain homeostasis under physiological conditions. Rather than fitting into binary activation states, recent high-dimensional transcriptomic and functional studies reveal that microglia exist along a dynamic and context-dependent continuum of states closely shaped by their microenvironment (Paolicelli et al., 2022). This updated framework better captures the remarkable heterogeneity and plasticity of microglia in both health and disease. This raises a critical question: how do activated microglia contribute to neuronal damage, particularly following CNS injury? While the mechanisms are multifactorial and not yet fully defined, recent research highlights the intricate crosstalk between microglial immune responses, neuroinflammation, and synaptic function.

Microglia and immune signaling pathways interact dynamically with synaptic structures under both normal and pathological conditions. Neuroinflammation, often referred to as a “double-edged sword,” plays a central role in the development and progression of neurodegenerative diseases. On one hand, it serves to eliminate harmful agents such as pathogens or cellular debris; on the other, it contributes to cytotoxicity and accelerates neuronal damage. Synaptic alterations are not only hallmark features of neurodegenerative diseases but also critical drivers of disease progression (Overk and Masliah, 2014). Inflammatory stimuli following neural injury can modulate synaptic efficacy and plasticity, leading to either long-term depression or potentiation depending on the molecular context (Stefano et al., 2025). Notably, chronic CNS inflammation is associated with sustained production of deleterious pro-inflammatory mediators that impair synaptic plasticity, ultimately contributing to maladaptive forms of synaptic remodeling (Cianciulli et al., 2022).

Under physiological conditions, microglia actively participate in the formation, elimination, and functional modulation of synapses. While some synaptic alterations are cell-autonomous, mounting evidence points to non-cell-autonomous mechanisms wherein microglia exert regulatory control over synaptic architecture and function. A key mechanism involves immune-related molecules, including cytokines and their receptors, that mediate communication between activated microglia and synaptic elements (Wake et al., 2019). These molecules are rapidly induced in response to disease, trauma, or infection and drive synaptic structural remodeling. In turn, altered synaptic states can further activate microglia, thereby creating a feed-forward loop that reinforces inflammation and disrupts synaptic homeostasis. This bidirectional interaction forms a mechanistic link between microglial overactivation and synaptic dysfunction.

The following sections examine current evidence that supports the contribution of microglia to inflammation-related synaptic dysregulation in neurodegenerative disorders.

## 3 Pro-inflammatory cytokines from activated microglia alter synaptic modification

**Figure 2:**
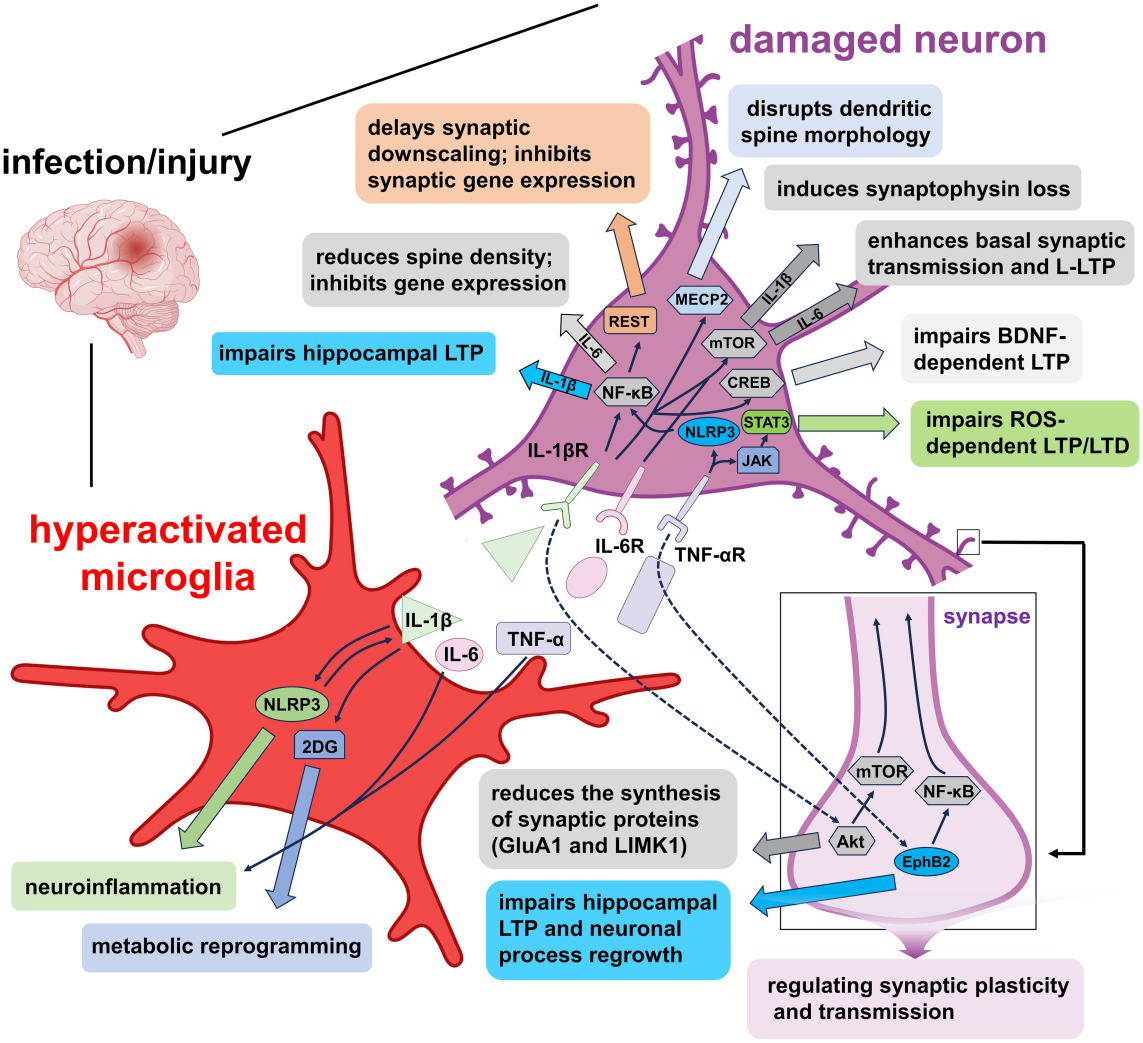
Mechanisms of pro-inflammatory factors-induced synaptic plasticity impairment under pathological conditions. Overactivated microglia release IL-1β, IL-6, and TNF-α, each disrupting synaptic function via distinct signaling pathways. IL-1β activates NLRP3 inflammasomes and the 2-DG metabolic pathway, promoting inflammation and metabolic reprogramming that further enhance microglial activation. Binding of IL-1β to IL-1βR engages signaling cascades such as NF-κB, NF-κB/REST, MECP2, mTOR, and CREB, leading to impaired synaptic downscaling, altered dendritic spine morphology, hippocampal LTP suppression, and reduced gene expression. Additionally, IL-1β disrupts synaptic protein synthesis through the Akt/mTOR pathway. IL-6 signaling via IL-6R facilitates basal synaptic transmission and L-LTP through mTOR activation. TNF-α, via TNF-αR, impairs hippocampal LTP and LTD through NLRP3/NF-κB and JAK/STAT3 pathways, while also affecting LTP and neuronal regeneration via the EphB2/NF-κB axis. Collectively, these cytokine-mediated mechanisms contribute to synaptic dysfunction and neurodegeneration by disrupting neuronal and microglial signaling homeostasis.

Neuroinflammation plays a pivotal role in the pathogenesis of neurodegenerative disorders. Elevated levels of pro-inflammatory cytokines, particularly IL-1β, IL-6, and TNF-*α*, have been observed in cognitive-related brain regions such as the hippocampus, underscoring their relevance in modulating synaptic structure and function (Yang et al., 2018). Upon encountering immune challenges, microglia become activated and secrete these cytokines, thereby contributing to synaptic dysregulation and the progression of neurodegenerative diseases (Figure 2). This section reviews the specific roles of pro-inflammatory cytokines in mediating synaptic modification in pathological conditions, emphasizing their mechanistic involvement in neuronal plasticity and degeneration.

### 3.1 IL-1β

IL-1β, a key pro-inflammatory cytokine generated via the proteolytic cleavage of pro-IL-1β, is central to both immune signaling and synaptic modulation. Under physiological conditions, IL-1β contributes to normal cognitive function and neural development by modulating synaptic plasticity, promoting memory consolidation, and regulating activity-dependent synaptic scaling (Yirmiya and Goshen, 2011). Activated microglia release IL-1β, which profoundly influences synaptic plasticity, including Hebbian plasticity and synaptic scaling (Stampanoni Bassi et al., 2020; Buffolo et al., 2021). Its elevated expression in the cortex, striatum, and hippocampus is closely associated with cognitive deficits (Yang et al., 2018; Zhao et al., 2019; Hoshino et al., 2021; Lin et al., 2019), prompting intensive investigations into its functional roles in neuroplasticity.

Impairment of long-term potentiation (LTP) by IL-1β has been widely documented as a mechanism underlying memory deficits. Rodent studies demonstrate that increased IL-1β levels suppress hippocampal LTP and impair learning and memory (Rizzo et al., 2018). Intracerebral injection of lipopolysaccharide (LPS) elevates IL-1β expression and disrupts LTP in the CA3-CA1 Schaffer collaterals (Golia et al., 2019; Yang et al., 2019; Li et al., 2024). Similar inhibitory effects are seen when IL-1β is applied to hippocampal slices (Hoshino et al., 2017; York et al., 2021) or isolated synaptosomes (Prieto et al., 2019). These deficits can be reversed by IL-1 receptor antagonists such as IL-1RA or anakinra (Hoshino et al., 2021; York et al., 2021; Prieto et al., 2019; Tong et al., 2018; Guo et al., 2020; Han et al., 2016). In delirium models, a negative correlation has been established between IL-1β levels and both LTP and hippocampus-dependent memory (Tanaka et al., 2018).

Mechanistically, IL-1β modulates neuroplasticity through multiple pathways. It activates immune signaling cascades such as the NOD-like receptor thermal protein domain associated protein 3 (NLRP3) inflammasome (Yu et al., 2023), impairs excitatory synapses in sepsis-associated encephalopathy (SAE) models (Moraes et al., 2022), and alters LTP via synaptic hyperexcitability in MS (Stampanoni Bassi et al., 2020). IL-1β interferes with N-methyl-D-aspartate receptor (NMDAR)-mediated calcium signaling, selectively impairing NMDAR-dependent LTP (Rizzo et al., 2018; Hoshino et al., 2017), and enhances Ca^2+^ influx in dorsal root ganglion neurons, contributing to pain processing (Noh et al., 2019). Anakinra has been shown to restore NMDAR-dependent hippocampal plasticity and reduce seizure susceptibility in prion disease models (Bertani et al., 2017).

IL-1β also influences synaptic scaling through RE1-silencing transcription factor (REST) activation, which delays synaptic downscaling at excitatory synapses (Buffolo et al., 2021). Additionally, IL-1β-mediated metabolic reprogramming of microglia impairs LTP, an effect reversible by glycolytic inhibition with 2-deoxyglucose (2DG) (York et al., 2021). In AD models, mitochondrial dynamics are disrupted by IL-1β through the activation of NF-κB and NLRP3 signaling pathways, resulting in Drp1-dependent fragmentation, excessive ROS production, and ATP depletion (Batista et al., 2021). It has been shown that these alterations impair synaptic energy metabolism and hippocampal LTP, thereby linking IL-1β-mediated mitochondrial dysfunction to cognitive decline (Batista et al., 2021). These findings suggest that IL-1β mediated bioenergetic imbalance represents a crucial pathogenic pathway in the progression of neurodegenerative diseases.

At the structural level, IL-1β impairs dendritic spine formation, alters spine morphology, and induces synaptic loss. These effects involve suppression of mechanistic target of rapamycin (mTOR) signaling and downregulation of key synaptic proteins such as GluA1 and LIMK1 (Prieto et al., 2019; Tong et al., 2018), activation of MECP2 and nuclear factor κB (NF-κB) pathways (Tomasoni et al., 2017; Fan et al., 2018), and inhibition of axonal growth via p38-MAPK (Han et al., 2017). IL-1β also promotes synaptic displacement through microglial engulfment mechanisms (You et al., 2024) and reduces synaptic protein expression via the PI3K/Akt/mTOR axis (Sheppard et al., 2019; Xiao et al., 2017). It induces tau hyperphosphorylation, which disrupts synaptic integrity (Ising et al., 2019; Jiang et al., 2021), and differentially modulates excitatory and inhibitory neurotransmission (Patel et al., 2019; Yan et al., 2019; Sung et al., 2017). Moreover, IL-1β-driven dysfunction involves the miR-142-3p/GLAST axis, underscoring the regulatory role of microglia in synaptic remodeling (Mandolesi et al., 2017).

Beyond direct synaptic effects, IL-1β interferes with BDNF signaling. It disrupts BDNF-Akt/CREB pathways, reducing LTP and contributing to synaptic degeneration (Li et al., 2024; Dong et al., 2018; Carlos et al., 2017). Knockdown of IL-1β restores inflammation-induced downregulation of BDNF and VGF (Li et al., 2017). Interestingly, BDNF can also stimulate IL-1β and TNF-*α* release from glial cells, creating a feedback loop that amplifies neuroinflammation (Ding et al., 2020). Depending on context, IL-1β-induced microglial activation may either exacerbate or mitigate neuronal injury by modulating BDNF release (Sung et al., 2017; Todd et al., 2019; Abd-El-Basset et al., 2020). Inhibition of IL-1β alleviates cognitive deficits in neuroinflammatory models (Taoro-Gonzalez et al., 2019). Collectively, these findings reveal IL-1β’s dual role in mediating microglial activation and neuroplasticity.

### 3.2 IL-6

IL-6, a pleiotropic pro-inflammatory cytokine primarily secreted by activated microglia and astrocytes, exerts multifaceted effects on various CNS cell types through both membrane-bound IL-6 receptor (IL-6Rα) and soluble IL-6 receptor (sIL-6R) pathways (Gruol et al., 2023). Beyond its pro-inflammatory roles, IL-6 also contributes to normal CNS physiology by modulating neuronal survival, synaptic function, and cognitive processes essential for brain development (Erta et al., 2012). Both IL-6 and its receptors are expressed in the hippocampus and play critical roles in CNS pathophysiology (Lin et al., 2019; Li et al., 2016; Willis et al., 2020; Onufriev et al., 2017). Under physiological conditions, IL-6 is present at low levels; however, its expression markedly increases during infections, injuries, and neurodegenerative or psychiatric disorders, implicating IL-6 in the regulation of hippocampus-dependent synaptic plasticity and memory.

Experimental evidence supports deleterious effects on synaptic function. Neonatal pro-inflammatory stress leads to impaired LTP and elevated IL-6 in rat hippocampal slices (Onufriev et al., 2017), while developmental IL-6 elevation disrupts connectivity through excessive excitatory synaptogenesis (Mirabella et al., 2021). *In vivo* studies further confirm that LPS-induced IL-6 elevation alters synaptic structure in the hippocampus (Lin et al., 2019), and IL-6 knockout in mice confers protection against age-related cognitive decline (Bialuk et al., 2020). In hippocampal slices from mice, IL-6 impairs LTP without affecting baseline synaptic excitability, an effect also observed in cerebrospinal fluid of relapsing–remitting multiple sclerosis (RR-MS) patients following paired associative stimulation (PAS) (Stampanoni Bassi et al., 2019).

IL-6 signaling is also implicated in amyloid-beta (Aβ) and tau pathologies. In AD mouse models (Tg2576, 3xTg-AD mice), inhibition of IL-6 trans-signaling reduces Aβ burden in both cortex and hippocampus (Escrig et al., 2019). Tau phosphorylation discussed in Section 3.1, is another key event influenced by IL-6. Chronic alcohol exposure and sevoflurane anesthesia elevate IL-6 levels, leading to tau hyperphosphorylation, mitochondrial dysfunction, and synaptic loss marked by reduced levels of postsynaptic density protein 95 (PSD-95), synaptophysin, N-cadherin, and total synapse number (Jiang et al., 2021; Zhang et al., 2020). These changes are accompanied by increased reactive oxygen species (ROS), decreased mitochondrial membrane potential (MMP), and reduced ATP levels, suggesting a mitochondrial pathway of IL-6-mediated neurotoxicity.

Microglial processing and release of tau may further drive IL-6 elevation and reactivation of microglia (Hopp et al., 2018), establishing a pathological loop. ROS-mediated synaptic pruning, although essential for physiological development, can become maladaptive under excessive mitochondrial ROS conditions, resulting in aberrant synapse elimination (Cobley, 2018; Sidlauskaite et al., 2018). IL-6 exacerbates this process by inducing mitochondrial dysfunction and oxidative stress, thereby contributing to synaptic degradation (Omoyinmi et al., 2016; Liu et al., 2017). These findings highlight the potential of IL-6-mitochondria-ROS interactions as therapeutic targets in neurodegenerative diseases.

In addition to structural changes, IL-6 modulates synaptic excitability and transmission, predominantly via trans-signaling (Rothaug et al., 2016). In genetically modified mice (GFAP-sgp130Fc, TG), inhibition of IL-6 trans-signaling enhances seizure susceptibility and increases synaptic excitability, evidenced by changes in paired-pulse ratios and prolonged seizures after pentylenetetrazole (PTZ) challenge (Cuevas-Olguin et al., 2017). IL-6 signaling also alters plasticity in a context-dependent manner: in social defeat models, it promotes resilience-associated plasticity but suppresses excitability in stress-susceptible animals (Esquivel-Rendón et al., 2019). Electrophysiological data from IL-6-overexpressing transgenic mice (IL-6 tg) demonstrate enhanced basal synaptic transmission and long-lasting LTP (L-LTP), yet rapamycin, a mTOR inhibitor, attenuates these effects, indicating a negative regulatory role of chronic IL-6 via mTOR signaling (Olde Engberink et al., 2017).

Behavioral outcomes also link IL-6 to synaptic dysfunction. Blocking IL-6 trans-signaling improves sociability in BTBR *T*^+^ *Itpr3^tf^* (BTBR) autism model mice, likely through enhanced cortical glutamate release (Wei et al., 2016), while cerebral IL-6 infusion impairs NMDA receptor-mediated synaptic currents and learning (Wang et al., 2019). Furthermore, IL-6 elevation contributes to depressive-like behaviors through reduced neuronal excitability and impaired GABAergic transmission (Roberts et al., 2019). In Fragile X syndrome (FXS) models, aberrant synapse formation and increased excitatory synapses are associated with elevated IL-6, emphasizing its role in excitatory/inhibitory imbalance (Krasovska and Doering, 2018).

Importantly, IL-6 may exert protective effects under specific pathological conditions (Stefano et al., 2025). In traumatic brain injury (TBI) models, IL-6 administration improves active place avoidance (APA) performance and promotes neurogenesis, suggesting a context-dependent neuroprotective role (Willis et al., 2020). Similarly, IL-6 produced by repopulated microglia aids in neuronal repair. These findings underscore the dual nature of IL-6 in the CNS and highlight the need for a nuanced understanding of its functions in neuroinflammation and synaptic modulation.

### 3.3 TNF-*α*

TNF-α is a pivotal pro-inflammatory cytokine present in both transmembrane (tmTNF-α) and soluble (sTNF-*α*) forms, the latter generated via TNF-α-converting enzyme (TACE). Its biological effects are primarily mediated through two receptors: TNFR1 and TNFR2. In the CNS, microglia serve as the principal source of TNF-*α*, though astrocytes and neurons also contribute. Moreover, microglial TNF-α has been shown to play a physiological role in regulating synaptic plasticity and supporting normal cognitive function under non-pathological conditions (Lewitus et al., 2016). Elevated TNF-*α* levels have been reported in various neurodegenerative and cognitive disorders, correlating with impaired hippocampal LTP, neuronal excitotoxicity, microglial activation, and neuroinflammation (Yang et al., 2019; Mashhadizadeh et al., 2017; Xu et al., 2019; Yang et al., 2021). Experimental studies demonstrate that exogenous TNF-*α* application reduces LTP in hippocampal slices and synaptosomes, while TNF-*α* neutralization or inhibition of microglial activation restores LTP and synaptic function (Prieto et al., 2019; Yang et al., 2021; Zhou and Bickler, 2017; Figueiredo et al., 2019). Furthermore, TNF-*α* mediates Aβ-induced LTP suppression via TNFR and downstream IKK/NF-κB signaling, as evidenced in *TREM2^R47H^* knock-in (KI) models where excessive TNF-*α* disrupted synaptic transmission and promoted Aβ production (Samidurai et al., 2018; Ren et al., 2020b).

TNF-*α* exhibits concentration-dependent bidirectional effects on synaptic plasticity: low concentrations facilitate synaptic potentiation, whereas elevated levels suppress it (Kleidonas et al., 2023). It preferentially targets excitatory synapses over inhibitory ones and regulates homeostatic synaptic scaling (Rizzo et al., 2018; Kleidonas and Vlachos, 2021). Studies in the mouse visual cortex revealed that TNF-*α* modulates dendritic spine size in a spatially restricted manner, aligning with synaptic scaling mechanisms. However, spine loss occurred independently of TNF-*α*, suggesting a role for Hebbian-like plasticity (Barnes et al., 2017). Collectively, these findings establish TNF-*α* as a crucial immune regulator of both Hebbian and homeostatic synaptic modifications.

Beyond classical plasticity, TNF-*α* also acts as a metaplasticity modulator, wherein previous synaptic activity shapes future plastic responses. This has implications for neurodegenerative pathologies. At Schaffer collateral-CA1 synapses, TNF-*α* was shown to modulate LTP in a concentration-dependent manner through mechanisms involving intracellular calcium stores and the actin-associated protein synaptopodin (Maggio and Vlachos, 2018). In APP/PS1 AD models, aberrant TNF-*α* release triggered pathological metaplasticity, impairing synaptic strengthening (Singh et al., 2019), further highlighting TNF-α’s central role in regulating synaptic adaptability.

TNF-α is also implicated in microglia-mediated synaptic pruning and structural remodeling. In primary cortical neurons, TNF-α (10 ng/mL) reduced neurite outgrowth and synaptic protein colocalization-effects reversed by tricyclic antidepressants (O'Neill et al., 2016). Interestingly, in human neuronal cultures under HIV-1-induced stress, TNF-α promoted neurite regrowth through Ephrin receptor B2 (EphB2)/NF-κB signaling, suggesting context-dependent duality in its actions (Pozniak et al., 2016). Additionally, TNF-α has been associated with neuropathic pain through modulation of synaptic strength and LTP. Intrathecal TNF-α mimicked vincristine-induced LTP at C-fiber synapses via upregulation of NR2B-containing NMDA receptors, while microglial inhibition or TNFR1 deletion attenuated TNF-α-driven synaptic changes and pain behaviors (Wigerblad et al., 2017; Xu et al., 2017; Liu et al., 2017). These data underscore the pathogenic synergy between TNF-α and microglia in both pain and synaptic dysfunction.

During neurodevelopment, TNF-α-related mechanisms also shape synaptic refinement. In Spinocerebellar Ataxia Type 1 (SCA1), NF-κB inhibition in Purkinje cells exacerbated motor deficits by interfering with microglial proliferation and TNF-α production, emphasizing the nuanced role of TNF-α signaling in neuronal circuitry formation (Ferro et al., 2018). Poly(I:C)-induced neuroinflammation further supports this, where increased TNF-α disrupted dendritic spine remodeling and perineuronal net (PNN) integrity, impairing synaptic and electrophysiological properties in hippocampal neurons via microglia-derived factors (Garre et al., 2017; Wegrzyn et al., 2021). In the auditory system, TNF-α-induced synaptopathy was reversible with etanercept, implicating TNF-α in broader sensory network dysfunctions (Hassan et al., 2017; Katsumi et al., 2019).

Emerging studies reveal that TNF-α facilitates pericyte-microglia interactions through NF-κB and tyrosine kinase (JAK)/STAT3 signaling, enhancing IL-6 release and sustaining neuroinflammation (Matsumoto et al., 2018). At the neuromuscular junction, TNF-α serves as a synaptotoxic regulator of synapse elimination (Fu et al., 2020). Moreover, in amyloid-based models of AD, TNF-*α* elevation promotes glutamatergic hyperactivity and the excitatory/inhibitory (E/I) imbalance in hippocampal circuits, with TNF-α blockade by XPro1595 preventing early synaptic deficits (Cavanagh et al., 2016) Similar hyperexcitability and LTP impairment were observed in TREM2^R47H^ KI rats, linking microglial dysfunction to TNF-*α*-mediated synaptic alterations (Ren et al., 2020a). Cocaine exposure and exogenous TNF-α infusion further support its role in glutamatergic transmission dysregulation and excitotoxicity in limbic regions (Wang et al., 2019; Lewitus et al., 2016).

Collectively, these studies establish TNF-α as a multifaceted modulator of synaptic plasticity and structure across development, health, and disease. Its involvement in synaptic transmission, pruning, metaplasticity, and neuroimmune interactions underscores its central role in neuroinflammatory-driven synaptic pathologies.

## 4 Receptors on activated microglia modulate synaptic modification

Microglia express a broad array of cytokine and chemokine receptors, enabling dynamic communication with surrounding neurons and glial cells beyond their traditional immune functions. Among these, cytokine/cytokine receptor signaling has emerged as a key modulator of synaptic structure and plasticity, linking immune activation to neuronal dysfunction. This section focuses on the role of microglia-expressed receptors, particularly under pathological conditions, in disrupting synaptic modification and contributing to neurodegenerative progression.

### 4.1 CD200/CD200R

CD200, a type I transmembrane glycoprotein, and its receptor CD200R are critical for maintaining microglial quiescence and immune homeostasis in the CNS. CD200 is primarily expressed on neurons, including in somas, axons, dendrites, and synapses, while CD200R is predominantly localized on microglia, with CD200 receptor 1 (CD200R1) showing the highest binding affinity among its receptor family members (Manich et al., 2019; Yang et al., 2023). CD200/CD200R signaling serves as an inhibitory axis that restrains microglial activation and suppresses excessive inflammatory responses. Multiple studies have demonstrated that activation of this pathway through the administration of CD200 fusion protein (CD200Fc), a CD200R1 agonist, alleviates neuroinflammatory responses and limits microglial activation in various disease models (Rabaneda-Lombarte et al., 2021; Wang et al., 2020; Hernangomez et al., 2016; Jiang et al., 2016). For instance, CD200Fc ameliorated Aβ-induced LTP deficits in the CA1 region of mouse hippocampal slices (Ngwa and Liu, 2019), while inhibition of CD200R impaired the anti-inflammatory phenotype of microglia under excitotoxic conditions (Yi et al., 2016). Clinically, downregulation of CD200R1 has been observed in the cerebrospinal fluid of patients with AD and delirium (Peters van Ton et al., 2020), and neuronal CD200 expression negatively correlates with hallmark AD pathologies including neurofibrillary tangles and amyloid plaques (Walker et al., 2017).

Functionally, CD200/CD200R signaling contributes to synaptic integrity by modulating microglial activity. CD200 engagement suppresses ATP release and microglial activation via ATP-sensitive potassium (KATP) channel opening, thereby reducing inflammatory cytokine secretion and protecting neurons (Ren et al., 2016). Notably, Wang et al. (2020) and Yi et al. (2016) reported that CD200R1 expression was significantly reduced in microglia following systemic LPS and *α*-synuclein challenge. CD200 knockout exacerbated microglial activation in the midbrain, leading to dopaminergic neuron loss in the substantia nigra, whereas CD200Fc administration reversed these changes. Interestingly, despite LPS inducing a nearly tenfold increase in cytokine release compared to α-synuclein, CD200R1 downregulation was comparable, CD200/CD200R1 signaling may influence microglia–neuron interactions beyond inflammation. Reduced CD200R1 expression in Parkinson’s models has been associated with microglial overactivation and synaptic dysfunction, supporting its role in maintaining synaptic stability and preventing maladaptive remodeling (Wang et al., 2020).

Supporting its synaptic role, CD200 has been identified in both pre- and post-synaptic compartments of excitatory synapses. In CD200-deficient mice, a significant reduction in synapse number was observed in the visual thalamus, implying a role in synaptic refinement (Loh et al., 2016). In a depression-like rat model, reduced CD200R expression in microglia was associated with aberrant synaptic plasticity (Wang et al., 2017). In APP/PS1 transgenic mice, hippocampal overexpression of CD200 via APP/PS1 mice, intrahippocampal injection of CD200 (AAV2/9-syn-CD200-mCherry) improved cognitive performance, restored synaptic function, and prevented synaptic loss by limiting microglial activation and secretion. Conversely, CD200 deletion exacerbated synaptic dysfunction and cognitive impairment (Feng et al., 2019). Similar findings were observed in a stroke model, where intracerebroventricular injection of CD200Fc restored synaptic integrity and motor function post- middle cerebral artery occlusion (MCAO), while CD200R antibody blockade worsened these outcomes (Sun et al., 2020). In a kainic acid-induced epilepsy model, upregulation of microglial CD200R by minocycline restored synaptic protein levels and dendritic morphology in the hippocampus, underscoring the receptor’s role in structural plasticity (Yang et al., 2023).

Interestingly, CD200 deficiency may also alter microglial phagocytic function. In Aβ-challenged CD200 knockout mice, enhanced phagocytosis and lysosomal activity reduced Aβ accumulation despite elevated microglial activation (Lyons et al., 2017). This paradoxical finding suggests a context-dependent role for CD200/CD200R signaling, wherein suppression of inflammatory activation must be balanced against potential impairments in Aβ clearance. Together, these studies establish the CD200/CD200R axis as a critical regulatory mechanism at the intersection of neuroinflammation and synaptic modification.

### 4.2 CX3CL1/CX3CR1

CX3CL1, also known as fractalkine (FKN), is a unique chemokine predominantly expressed by neurons and inducible in astrocytes under pro-inflammatory conditions (Luo et al., 2019). It exists in both membrane-bound and soluble forms, both of which are biologically active and signal through CX3CR1, a receptor selectively expressed on microglia in the CNS (Luo et al., 2019). The CX3CL1-CX3CR1 axis represents a critical molecular interface for neuron–microglia communication, modulating microglial activation and synaptic function in health and disease.

Recent evidence highlights the multifaceted role of this signaling pathway in regulating neuroinflammatory responses and synaptic remodeling (Wang et al., 2023). For instance, reduced CX3CL1 levels have been observed in schizophrenia, accompanied by strong correlations between CX3CR1 expression and presynaptic protein SNAP-25 (Hill et al., 2020). In the auditory system, CX3CR1 mutations alter synaptic organization, particularly in regions involved in complex sensory processing (Milinkeviciute et al., 2021). In PD models, CX3CR1 is downregulated at early stages and is sensitive to LPS or *α*-synuclein in primary microglia, indicating that inflammatory triggers dynamically regulate its expression (Wang et al., 2020). In the hippocampus, CX3CR1 maintains normal glutamatergic transmission; its disruption leads to impaired synaptic function and abnormal synapse morphology (Basilico et al., 2021).

Ischemic brain injury models further demonstrate that CX3CL1 downregulation promotes microglial-mediated inflammation and neuronal autophagy (Zhu et al., 2019). In parallel, CX3CR1 modulates microglial morphology during this process, indicating its importance in inflammatory microglial phenotypes (van der Maten et al., 2017). In AD models (APP/PS1), mesenchymal stem cells (MSCs) overexpressing CX3CL1 reduced TNF-α levels and upregulated synaptic proteins, though cognitive benefits were observed only when CX3CL1 was co-expressed with Wnt3a (Li et al., 2020). Membrane-bound CX3CL1 also appears to maintain microglia in a surveying, non-reactive state. CX3CR1-deficient (CX3CR1^−/−^) mice exhibit excessive microglial activation, increased extracellular matrix deposition, and impaired synaptic integration in dentate gyrus neurons, along with reductions in dendritic spine density, axonal terminal area, and overall synaptic function. Behaviorally, female CX3CR1^−/−^ mice display hyperactivity, reduced anxiety, and depressive-like behaviors (Bolós et al., 2018).

Further studies confirm that CX3CR1 is essential for activity-dependent synaptic plasticity. In the motor cortex, its absence disrupts learning and synaptic remodeling under direct current stimulation (Gellner et al., 2023). In the mouse barrel cortex, CX3CR1/CX3CL1/ADAM10 signaling mediates synaptic engulfment by microglia during whisker deprivation, indicating its involvement in experience-dependent synaptic elimination (Gunner et al., 2019). Similarly, the pathway contributes to synapse pruning and the maintenance of newly integrated neurons (Reshef et al., 2017), supporting its fundamental role in homeostatic synaptic regulation.

However, CX3CL1/CX3CR1 signaling can exert context-dependent and even opposing effects on synaptic plasticity. In AD models, CX3CR1 deletion enhances Aβ clearance in a gene dose-dependent manner (Merino et al., 2016) and appears to protect synapses from stress-induced (Milior et al., 2016; Schubert et al., 2018) or HIV gp120-induced neuronal injury (Ru et al., 2019). Elevated CSF CX3CL1 levels in delirium and AD patients are associated with decreased synapse-associated proteins (Peters van Ton et al., 2020), suggesting disease-specific alterations in signaling dynamics. Mechanistically, CX3CL1 inhibits CA1 hippocampal LTP via activation of adenosine A3 receptors (A3R) (Poniatowski et al., 2017). Under hypoxic conditions, CX3CL1/CX3CR1 signaling promotes microglial phagocytic activity, accelerating synaptic loss (Wang et al., 2023).

Interestingly, the effects of CX3CL1/CX3CR1 signaling appear to be region- and disease stage-specific. For example, in the visual system, CX3CL1 deletion does not affect microglial morphology or synaptic turnover (Lowery et al., 2017), while in stroke models, CX3CR1 deficiency does not alter brain injury outcomes (van der Maten et al., 2017). Similarly, in CA3 pyramidal neurons, GABAergic transmission remains unchanged in CX3CR1^−/−^ mice, although a notable reduction in giant depolarizing potential (GDP) frequency is observed (Bertot et al., 2019). Collectively, these findings underscore the complexity of the CX3CL1/CX3CR1 axis, which exerts diverse, and sometimes contradictory, effects on synaptic structure and plasticity depending on the brain region, pathological condition, and cellular context.

### 4.3 CSF1/CSF1R

CSF1, also known as macrophage colony-stimulating factor (MCSF), is a cytokine essential for the development and maintenance of cells in the mononuclear phagocyte system. Its receptor, CSF1R (c-Fms/CD115), belongs to the type III receptor tyrosine kinase family. In the CNS, CSF1 is mainly expressed by neurons, whereas CSF1R is selectively expressed on microglia (Chitu et al., 2016). CSF1/CSF1R signaling governs multiple microglial processes, including migration, proliferation, survival, differentiation, and polarization, and plays a fundamental role in regulating neuroinflammation. However, excessive activation of this pathway has been linked to pathological neuroinflammatory states, thereby positioning CSF1R inhibition as a potential therapeutic strategy (El-Gamal et al., 2018).

In a lumbar disc puncture model using CX3CR1^GFP/+^ mice, upregulation of CSF1/CSF1R signaling intensified microglial activation after disc degeneration (Yang et al., 2018), suggesting a link between this axis and maladaptive neuroplasticity. CSF1R inhibitors such as PLX5622 suppress microglial activation and neuroinflammation while preserving synaptic structure and function. Low-dose PLX5622 has been shown to prevent sepsis induced synaptic loss and cognitive impairment, underscoring the role of CSF1R signaling in inflammation related synaptic remodeling (Mein et al., 2023). Guan et al. (2016) demonstrated that deletion of CSF1 from sensory neurons reduced microglia-mediated activation and mechanical hypersensitivity after nerve injury, likely through disruption of the CSF1R/DAP12 pathway. Conversely, exogenous CSF1 administration had the opposite effect. Similar microglial responses were observed in neuropathic pain (Okubo et al., 2016) and autoimmune encephalomyelitis models (Gushchina et al., 2018), in which CSF1 expression correlated with microglial proliferation and enhanced neuroinflammation. Intrathecal injection of anti-CSF1 antibodies also reversed high-frequency stimulation (HFS)-induced spinal LTP at C-fiber synapses and attenuated CSF1R expression and microglial activation, thereby highlighting the contribution of this pathway to microglia-driven synaptic plasticity (Zhou et al., 2019).

Beyond pain and inflammatory disorders, CSF1/CSF1R signaling plays a critical role in microglia-mediated synaptic pruning. In chronic unpredictable stress (CUS) models of cognitive impairment, increased CSF1/CSF1R activity drove microglial engulfment of neuronal components and spine loss in the medial prefrontal cortex (mPFC). These effects were reversed by neuronal CSF1 knockdown (Wohleb et al., 2018), glucocorticoid receptor antagonism (RU486) (Horchar and Wohleb, 2019), or diazepam treatment (Bollinger et al., 2020), indicating that CSF1-dependent microglial remodeling contributes to stress-induced behavioral and synaptic deficits. In SAE models, inhibition of CSF1R by PLX5622 reduced C1q-tagged synapses and microglial phagocytosis, providing further evidence that this pathway regulates synaptic elimination under inflammatory conditions (Chung et al., 2023). Prolonged CSF1R blockade using small molecules such as pexidartinib has been shown to attenuate neuroinflammatory damage and stabilize synaptic integrity, potentially facilitating neuronal recovery (Weyer et al., 2024). Similar neuroprotective effects were reported in models of MS, where CSF1R inhibition prevented synaptic loss in the context of cortical inflammation (Ding et al., 2021).

Emerging data also suggest a pathogenic role for CSF1 in early-stage AD (Klein et al., 2020). In APP/PS1 mice, administration of the tyrosine kinase inhibitor (GW2580) improved memory and behavior by suppressing microglial proliferation and synaptic degeneration (Olmos-Alonso et al., 2016). Short-term CSF1R inhibition in 5xFAD mice led to reduced neuroinflammation and enhanced autophagy in residual microglia, which was associated with mitigation of synaptic loss (Kodali et al., 2024). Interestingly, withdrawal of CSF1R inhibitors facilitated microglial repopulation, which promoted brain recovery and reduced inflammation following injury (Rice et al., 2017). Similar observations were made in TBI models, where repopulated microglia restored synaptic and neuronal function while reducing long-term inflammation (Wang et al., 2022). Furthermore, neuronal CSF1 was found to modulate depression-like behaviors via microglial STAT3-dependent regulation of synaptic transmission (Kwon et al., 2017). In models of neuropathic pain, CSF1 increased excitatory input to excitatory neurons in a BDNF-dependent manner and concurrently reduced excitatory input to inhibitory neurons via a BDNF-independent mechanism, thereby shifting the excitatory/inhibitory balance (Boakye et al., 2019).

CSF1R signaling is also developmentally regulated. In the olfactory bulb, microglial depletion via PLX5622 reduced both synapse formation and elimination in young, but not mature, adult-born granule cells (abGCs), indicating a time-specific requirement for microglia in synaptic remodeling (Reshef et al., 2017). Similarly, in a retinitis pigmentosa (RP) model, treatment with the CSF1R inhibitor PLX3397 reduced microglia-mediated synaptic pruning (He et al., 2019). Collectively, these findings underscore the dual and context-dependent role of CSF1/CSF1R signaling in shaping synaptic architecture under both physiological and pathological conditions.

### 4.4 IFN-*γ*/IFN-γR

IFN-γ, the sole type II interferon, is a soluble pro-inflammatory cytokine primarily produced by T helper cell type 1 (Th1) lymphocytes, CD8^+^ cytotoxic T cells, natural killer (NK) cells, and microglia within the CNS (Monteiro et al., 2017). It exerts its biological effects via binding to the IFNGR1/IFNGR2 receptor complex expressed on both microglia and neurons (Monteiro et al., 2017). IFN-γ is a potent activator of microglia, inducing phenotypic polarization, receptor upregulation, morphological changes, and elevated expression of inflammatory mediators, including autocrine IFN-γ release. This, in turn, initiates transcriptional programs that drive classical activation (Vergara et al., 2019; Lewen et al., 2020; Ta et al., 2019).

Functionally, IFN-γ-activated microglia modulate synaptic integrity and plasticity. In hippocampal slice cultures, IFN-γ stimulation enhances microglial activation and proliferation, accompanied by morphological remodeling and increased production of nitric oxide and pro-inflammatory cytokines (Lewen et al., 2020; Ta et al., 2019). These changes underlie heightened synaptic pruning and impaired neuroplasticity. For instance, systemic delivery of IFN-γ upregulates major histocompatibility complex class I (MHCI) in the mPFC, promoting synapse elimination (Sobue et al., 2018). IFN-γ-deficient mice display improved hippocampal synaptic architecture, neuronal morphology, and cognitive performance, suggesting that basal IFN-γ negatively regulates synaptic plasticity (Monteiro et al., 2016). Mechanistically, IFN-γ activates STAT1 signaling in hippocampal microglia, priming them for excessive pruning and disrupting social memory via aberrant microglia–neuron interactions (He et al., 2024).

Elevated IFN-γ has also been implicated in synaptic degeneration in disease models. In experimental autoimmune encephalomyelitis (EAE), neutralization of IFN-γ alleviated dendritic spine loss in the cortex (Huang et al., 2021). Similarly, genetic ablation of CD8 + T cells or microglial IFN-γ signaling conferred resistance to virus-induced synapse loss and neuronal apoptosis in West Nile virus (WNV) and Zika virus (ZIKV) models (Garber et al., 2019). In PD models, IFN-γ exacerbates neuronal susceptibility by enhancing the pathological leucine-rich repeat kinase 2 (LRRK2)-G2019S pathway, thereby impairing Akt signaling and nuclear factor of activated T-cells (NFAT) activation in both neurons and microglia (Panagiotakopoulou et al., 2020). In the rostral ventrolateral medulla (RVLM), IFN-γ deficiency compromised microglial synaptic engulfment, highlighting its role in modulating synaptic density and excitability with implications for hypertension pathophysiology (Tong et al., 2023).

Beyond structural remodeling, IFN-γ disrupts E/I balance by altering neurotransmission. In neuropathic pain, exogenous IFN-γ enhances synaptic transmission from C-fibers to spinal lamina I neurons through microglial activation (Reischer et al., 2020). In MS models, IFN-γ gene delivery promotes microglia-driven glutamate release and paranodal elongation—reversible with NMDA receptor antagonists (Gallego-Delgado et al., 2020). Similarly, in Toxoplasma gondii-infected mice, IFN-γ-primed microglia impair synaptic transmission and neural oscillations, aggravating cognitive deficits. Co-infection with Heligmosomoides polygyrus further amplifies Th1 cytokine production and suppresses synaptic markers such as EAAT2 and GABAA*α*1, effects mitigated by IFN-γ neutralization, suggesting that IFN-γ drives neuroinflammation-associated synaptic dysfunction (Kann et al., 2022; French et al., 2019).

Despite its deleterious roles, IFN-γ also exerts context-dependent neuroprotective functions. In models of immune dysfunction, IFN-γ enhances GABAergic currents in the prefrontal cortex (PFC) neurons, preventing hyperexcitability and preserving social behavior (Filiano et al., 2016). In AD models, IFN-γ facilitates amyloid-*β* clearance by restoring autophagy in microglia (He et al., 2020). Environmental enrichment–induced synaptic benefits, including elevated dendritic spine density and LTP, are abrogated by IFN-γ blockade, further supporting its context-specific modulatory role (Qi et al., 2018). Thus, IFN-γ emerges as a double-edged cytokine—detrimental when dysregulated, yet potentially protective under physiological or therapeutic conditions (John and Darcy, 2016).

## 5 Microglial regulation of the effects of BDNF and TREM2 on synaptic modification

### 5.1 BDNF

BDNF, a member of the neurotrophin family, plays multifaceted roles in the CNS, contributing to neuronal maturation, circuit refinement, and synaptic modulation. While predominantly produced by neurons and astrocytes, BDNF is also synthesized by microglia, where it exhibits anti-inflammatory properties and contributes to the maintenance of CNS homeostasis (Charlton et al., 2023). Accumulating evidence indicates that BDNF is critically involved in synaptic remodeling during pathological conditions, particularly via its facilitation of hippocampal LTP. Reduced BDNF levels are associated with impaired LTP (Golia et al., 2019), whereas exogenous BDNF administration restores synaptic plasticity in memory-deficient models (White et al., 2016).

The mechanisms underlying BDNF-mediated LTP involve multiple signaling pathways and synaptic regulators. These include activation of cannabinoid type 1 receptors (CB1Rs) (Maglio et al., 2018), upregulation of the RNA-binding protein RBFOX1 (Tomassoni-Ardori et al., 2019), enhanced expression of activity-regulated cytoskeleton-associated protein (Arc/Arg3.1) (Kuipers et al., 2016), and engagement of mature BDNF (mBDNF)/TrkB/CREB signaling cascades (Zhou et al., 2019; Garad et al., 2021; Pan et al., 2019). Moreover, peri-synaptic glial recycling of BDNF has been identified as a critical process modulating its availability at synaptic sites (Vignoli et al., 2016). Microglia-derived BDNF is implicated in both physiological and pathological synaptic plasticity, contributing to neonatal incision-induced spinal LTP (Ding et al., 2018) and cocaine-induced adaptations (Cotto et al., 2018), as well as promoting neuroplasticity and stress resilience (Prowse and Hayley, 2021).

Notably, dysregulation of BDNF signaling can also contribute to neuroinflammation and synaptic impairment. Activation of P2X4 receptors on microglia reduces hippocampal BDNF levels, exacerbates inflammation, and impairs late-phase LTP (L-LTP) (Tanaka et al., 2018; Wei et al., 2024). Conversely, enhanced BDNF/TrkB signaling can shift microglial phenotypes toward anti-inflammatory states and protect against neuronal loss (Hsu et al., 2020). However, BDNF produced by microglia has also been shown to promote pro-inflammatory responses, including the release of cytokines such as IL-1*β* and TNF-*α* (Ding et al., 2020). This bidirectional role emphasizes the importance of context-dependent regulation. As noted in Sections 3.1 and 3.3, targeting the interaction between BDNF and microglial-derived cytokines may offer therapeutic potential in neurodegenerative conditions.

At the synaptic level, BDNF enhances NMDAR function by increasing the ratio of GluN2B-containing subunits, a process dependent on proline-rich tyrosine kinase 2 (Pyk2) and heterogeneous nuclear ribonucleoprotein K (hnRNP K) activity (Afonso et al., 2019). It also regulates dendritic spine structure, reversing chronic unpredictable mild stress (CUMS)-induced spine loss and behavioral deficits in rodents (Park et al., 2016; Qiao et al., 2017). In Nhe5-deficient mice, upregulation of BDNF/TrkB signaling correlates with improved cognition (Chen et al., 2017). While numerous studies associate BDNF with changes in dendritic spine morphology, causal relationships, particularly those involving microglial modulation, remain incompletely characterized.

BDNF further modulates microglial activity by preventing excessive synaptic pruning and limiting microglial proliferation in kainic acid-induced injury models (Onodera et al., 2021). Interestingly, in the developing cerebellum, retrograde BDNF–TrkB signaling from Purkinje cells promotes climbing fiber synapse elimination, indicating regional and developmental specificity (Choo et al., 2017). BDNF also regulates the balance between excitatory and inhibitory neurotransmission, with age-dependent alterations in PFC linked to deficits in GABAergic signaling due to disrupted BDNF/NTRK2 pathways (Oh et al., 2016; Meis et al., 2019). In neuropathic pain, microglia-derived BDNF contributes to excitatory/inhibitory imbalance via TrkB and p75 neurotrophin receptor (p75^NTR^), enhancing dorsal horn excitability (Boakye et al., 2019). Similar interactions involving KCC2 regulation are implicated in ischemia-induced plasticity changes (Cramer et al., 2022). Furthermore, crosstalk between BDNF/TrkB and the endocannabinoid system (ECS) plays a significant role in modulating microglial responses and synaptic strength (Maglio et al., 2018; Pan et al., 2019; Yeh et al., 2017; Wu et al., 2020; Gangarossa et al., 2020).

Collectively, these findings establish BDNF as a critical, yet context-dependent, modulator of microglia–neuron interactions, synaptic architecture, and neurotransmission. Its dual role in promoting neuroplasticity and contributing to inflammation underscores the importance of understanding BDNF signaling in a cell-type- and disease-specific manner.

### 5.2 TREM2

TREM2 is a microglia-specific type I transmembrane receptor that plays a crucial role in regulating microglial activity, particularly in synaptic pruning and the pathophysiology of neurodegenerative diseases. TREM2 activation promotes the anti-inflammatory microglial phenotype, thereby enhancing phagocytic function, attenuating neuroinflammation, and mitigating synaptic and neuronal loss under pathological conditions (Jiang et al., 2016; Ruganzu et al., 2021; Zhang et al., 2018; Zhang et al., 2017; Jiang et al., 2018; Fracassi et al., 2022). In addition, TREM2 signaling restrains microglial overactivation induced by chronic neuroinflammation (Chen et al., 2020; Krasemann et al., 2017; Ruganzu et al., 2022), and has been implicated in the regulation of neuroplasticity (Raha et al., 2017). Interestingly, TREM2 deficiency enhances LTP without affecting basal synaptic transmission in aged mice (Qu and Li, 2020). Moreover, the AD-associated R47H mutation demonstrates an age-dependent impact on LTP: it initially prevents LTP deficits but later exacerbates them, suggesting a temporally dynamic role of TREM2 in synaptic plasticity (Tran et al., 2023).

In addition to its role in AD, TREM2 modulates microglial activity in other neurodegenerative diseases, including PD and ALS. In PD, TREM2 expression is upregulated in activated microglia surrounding dopaminergic neurons, enhancing phagocytosis and suppressing the excessive release of pro-inflammatory cytokines (Zhang et al., 2018). It has been shown that TREM2 signaling inhibits NLRP3 inflammasome activation and pyroptosis, thereby reducing dopaminergic neuron loss in MPTP induced models. Conversely, TREM2 deficiency results in increased IL-1β and TNF-*α* production, exacerbating neuroinflammation (Huang et al., 2023). In ALS, TREM2 expression exhibits stage-dependent changes, with upregulation observed in the early and mid-stages of the disease, correlating with a reactive yet neuroprotective microglial phenotype (Jericó et al., 2023). Overall, emerging evidence suggests that TREM2 acts as a key regulatory hub linking innate immune receptor signaling with cytokine-mediated inflammatory pathways, thereby maintaining microglial homeostasis and modulating neuroinflammatory and synaptic outcomes in PD, ALS, and other neurodegenerative diseases (Yeh et al., 2017).

TREM2 also contributes to synaptic pruning. Several studies have shown that TREM2 facilitates microglial engulfment of synaptic components (Fracassi et al., 2022; McQuade et al., 2020; Filipello et al., 2018), with the R47H mutation promoting excessive synaptosome phagocytosis, which may result in abnormal synaptic elimination and accelerated AD progression (Popescu et al., 2022; Das et al., 2023; Penney et al., 2023; Gratuze et al., 2020). In APP/PS1 transgenic mice, early TREM2 deficiency appears to prevent synaptic loss by reducing microglial phagocytic activity, whereas at later stages, its absence impairs amyloid clearance and worsens synaptic dysfunction (Ruganzu et al., 2022; Sheng et al., 2019). Similarly, in a dual-pathology AD model incorporating both β-amyloid and tau, TREM2 deletion disrupts microglial synapse phagocytosis (Dejanovic et al., 2022). External factors, such as chronic alcohol exposure, have also been shown to upregulate TREM2 expression, leading to increased synaptic elimination that correlates with memory impairments and reduced synaptic density (Lan et al., 2022). Mechanistically, TREM2-mediated synaptic remodeling involves p38 MAPK activation, complement pathway engagement, and interactions with phosphatidylserine on neuronal membranes (Tian et al., 2024; Rueda-Carrasco et al., 2023; Zhong et al., 2023; Meng et al., 2024). Notably, astrocytic uptake of synapses may also contribute to these effects, suggesting that TREM2’s role extends beyond direct microglia-synapse interactions (Jay et al., 2019).

Beyond structural remodeling, TREM2 influences neurotransmission. Its deficiency has been associated with elevated excitatory synaptic activity during early development (Filipello et al., 2018). In the context of AD, TREM2 suppresses glutamatergic transmission at early stages but appears to facilitate it at later stages (Sheng et al., 2019). The R47H mutation is further implicated in reducing GABAergic transmission via TNF-*α* upregulation, aggravating glutamate-induced excitotoxicity (Ren et al., 2020b; Ren et al., 2020a). These observations indicate that TREM2 exerts stage-dependent and context-specific effects on both excitatory and inhibitory transmission.

From a therapeutic standpoint, interventions targeting TREM2 must account for its multifaceted roles in phagocytosis, inflammation resolution, and apoptosis inhibition. While augmenting TREM2 signaling may offer benefits in certain neurodegenerative contexts, inappropriate modulation could inadvertently trigger detrimental pro-inflammatory cascades (Akhter et al., 2021; Kobayashi et al., 2016). Paradoxically, TREM2 loss has been associated with resistance to age-related synaptic and cognitive decline (Qu and Li, 2020), underscoring the nuanced and context-sensitive nature of its functions.

Collectively, TREM2 acts as a dynamic modulator of microglial behavior, synaptic remodeling, and neurotransmission. Its impact is highly dependent on developmental stage, pathological context, and mutation status, emphasizing the importance of precise therapeutic strategies when targeting TREM2-associated pathways.

## 6 Concluding remarks

Our understanding of microglia, the brain’s resident immune cells, continues to evolve. Once recognized primarily for their role in immune surveillance, microglia are now understood to play essential roles in maintaining neural homeostasis and regulating synaptic architecture. Increasing evidence suggests that hyperactivation of microglia, often accompanied by sustained neuroinflammatory responses, contributes significantly to the pathogenesis of neurological disorders. This review has highlighted recent advances linking dysregulated microglia-mediated synaptic remodeling and aberrant expression of pro-inflammatory cytokines and receptors to both neurodevelopmental and neurodegenerative diseases.

**Figure 3:**
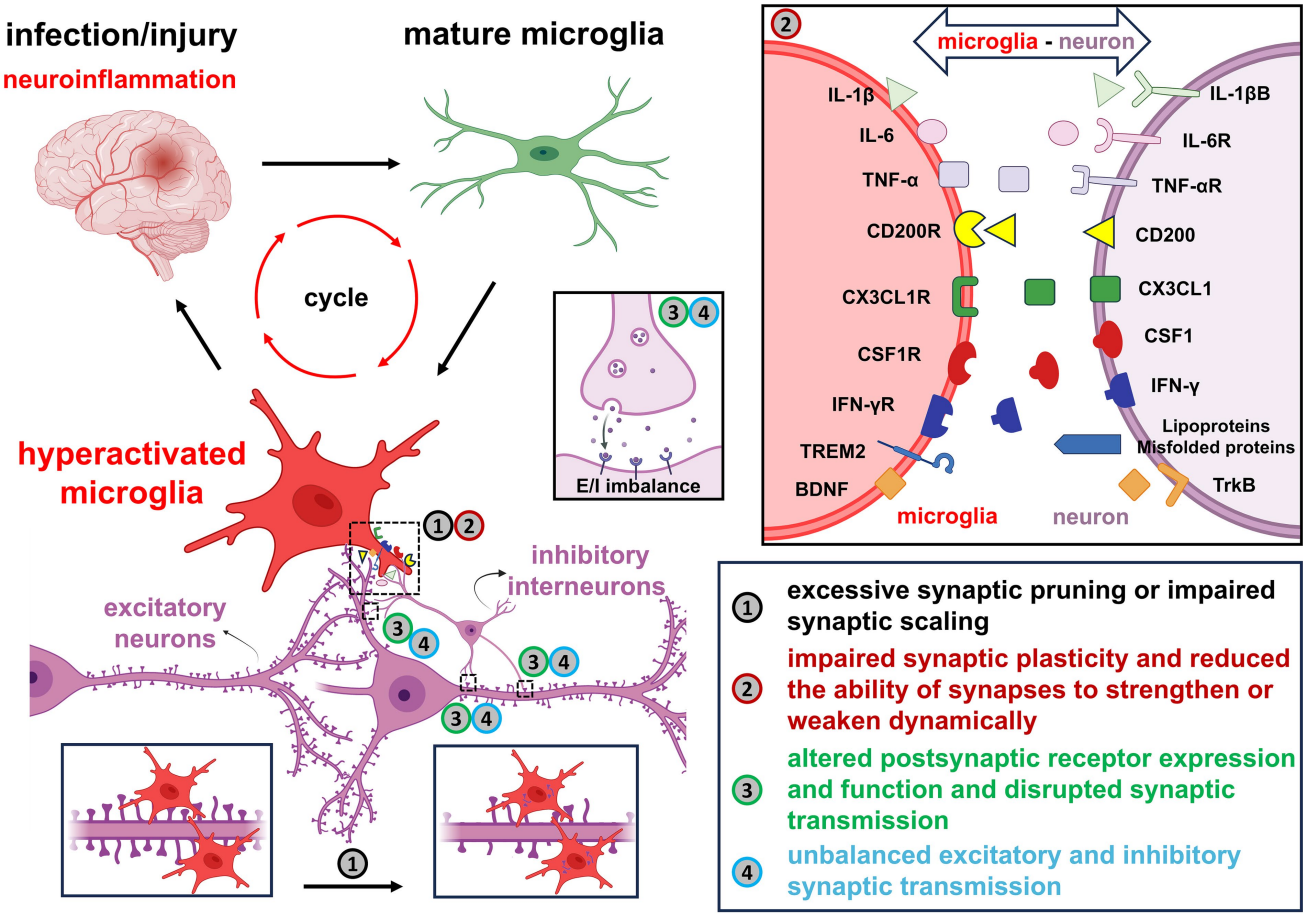
A summary of the impact of overactivated microglia on neuronal synapses under neuroinflammatory conditions. Following infection or injury, microglia transition into hyperactivated pro-inflammatory states and secrete cytokines such as IL-1β, IL-6, TNF-α and IFN-γ, which exacerbate neuroinflammation and contribute to synaptic impairment. Disruption of key neuron–microglia ligand-receptor interactions, such as CD200/CD200R, CX3CL1/CX3CL1R, CSF1/CSF1R, Lipoproteins/Misfolded proteins/TREM2 and BDNF/TrkB, leads to dysregulated synaptic homeostasis. The resulting microglial activation promotes excessive synaptic pruning, impaired synaptic scaling, abnormal receptor expression, and altered synaptic transmission. These changes ultimately disturb the excitatory-inhibitory balance and compromise overall neuronal function.

Following CNS injury, activated microglia release pro-inflammatory mediators, initiating an inflammatory cascade that induces structural and functional synaptic alterations, ultimately leading to cognitive and behavioral impairments (Figure 3 and Table 1). Notably, such microglia-driven synaptic disruption may aggravate secondary injury, thereby amplifying neurodegeneration. Consequently, timely and effective suppression of harmful microglial responses is crucial. Nevertheless, microglial responses are highly dynamic and context-dependent, encompassing a spectrum of functional states ranging from pro-inflammatory to restorative and phagocytic profiles. These diverse and adaptable responses can promote either neuroprotection or neurotoxicity depending on the surrounding microenvironment and disease stage. Hence, therapeutic interventions targeting microglia must be carefully designed to minimize adverse effects. Beyond their intrinsic roles, microglia-mediated cytokine signaling orchestrates complex interactions among astrocytes, oligodendrocytes, and peripheral immune cells, collectively shaping synaptic remodeling and influencing the progression of neurodegeneration. A deeper understanding of this intercellular communication network may reveal regulatory mechanisms and therapeutic targets to mitigate microglia-driven neuroinflammatory and neurodegenerative processes.

**Table 1:** Negative regulation of synaptic modifications and behavioral correlates by microglial pro-inflammatory factors/receptors.

Cytokines/receptors	Synaptic modifications	Associated signaling pathways	Behaviors phenotypes
Elevated IL-1β levels	Inhibits LTP (Stampanoni Bassi et al., 2020; Hoshino et al., 2021; Hoshino et al., 2017; York et al., 2021; Prieto et al., 2019; Guo et al., 2020; Han et al., 2016; Bertani et al., 2017); induces synaptic downscaling (Buffolo et al., 2021); impairs metaplasticity (Bertani et al., 2017); promotes hyperexcitability and excitatory transmission via the glutamatergic system (Stampanoni Bassi et al., 2020; Hoshino et al., 2021; Noh et al., 2019; Yan et al., 2019; Sung et al., 2017; Mandolesi et al., 2017); enhances glutamate release (Sung et al., 2017); induces inhibitory transmission via glutamatergic signaling (Buffolo et al., 2021; Prieto et al., 2019; Tong et al., 2018; Bertani et al., 2017; Taoro-Gonzalez et al., 2019); bidirectionally modulates GABAergic transmission (Patel et al., 2019); reduces dendritic spine density (Fan et al., 2018); causes synapse loss (Yu et al., 2023; Batista et al., 2021); disrupts axon development (Han et al., 2017); induces synaptic displacement (You et al., 2024)	REST/NRSF (Buffolo et al., 2021); PI3K/Akt/mTOR (Prieto et al., 2019; Han et al., 2016; Xiao et al., 2017); P2X7R/NLRP3/IL-1β (Guo et al., 2020; Yu et al., 2023); mBDNF/TrkB (Tanaka et al., 2018); sphingomyelinase/ceramide/Src (Tong et al., 2018); IL-1β/NF-κB (Fan et al., 2018); p38/MAPK (Han et al., 2017); p-p38/iNOS/NO (Sung et al., 2017); IL-1β/miR-142-3p/GLAST (Mandolesi et al., 2017); ROS-NF-κB (Li et al., 2024)	Depression-like behaviors (Fan et al., 2018; Carlos et al., 2017); anxiety-like behaviors (Carlos et al., 2017); nociceptive behaviors (Sung et al., 2017); impaired learning and memory (Li et al., 2024; You et al., 2024; Carlos et al., 2017; Taoro-Gonzalez et al., 2019)
Elevated IL-6 levels	Inhibits LTP (Stampanoni Bassi et al., 2019); enhances late-phase LTP (Olde Engberink et al., 2017); modulates excitatory transmission via glutamatergic signaling and neurotransmitter release (Mirabella et al., 2021; Esquivel-Rendón et al., 2019; Olde Engberink et al., 2017; Krasovska and Doering, 2018); regulates inhibitory transmission via glutamatergic and GABAergic systems (Cuevas-Olguin et al., 2017; Esquivel-Rendón et al., 2019; Wang et al., 2019); decreases glutamate release (Wei et al., 2016) and ATP levels (Zhang et al., 2020; Omoyinmi et al., 2016); causes abnormal dendritic spine morphology (Krasovska and Doering, 2018); leads to synapse loss (Zhang et al., 2020)	IL-6/STAT3/Bcl-2 (Bialuk et al., 2020); IL-6/mTOR (Olde Engberink et al., 2017); STAT3/RGS4 (Mirabella et al., 2021)	Depression-like behaviors (Esquivel-Rendón et al., 2019); anxiety-like behaviors (Escrig et al., 2019); autism-like behaviors (Wei et al., 2016); impaired learning and memory (Escrig et al., 2019; Wang et al., 2019)
Elevated TNF-α levels	Inhibits LTP (Prieto et al., 2019; Yang et al., 2021; Zhou and Bickler, 2017; Figueiredo et al., 2019; Samidurai et al., 2018; Ren et al., 2020b; Singh et al., 2019; Liu et al., 2017; Ren et al., 2020a); promotes LTP (Wigerblad et al., 2017; Xu et al., 2017; Liu et al., 2017; Cavanagh et al., 2016); induces synaptic scaling (Barnes et al., 2017); alters metaplasticity (Maggio and Vlachos, 2018; Singh et al., 2019); enhances excitatory and inhibitory transmission via glutamatergic/GABAergic signaling (Wang et al., 2019; Ren et al., 2020b; Kleidonas et al., 2023; Wigerblad et al., 2017; Liu et al., 2017; Wegrzyn et al., 2021; Cavanagh et al., 2016; Ren et al., 2020a); reduces dendritic spine length and density (Liu et al., 2017; Garre et al., 2017); causes synaptic atrophy/loss (Figueiredo et al., 2019; O'Neill et al., 2016); impairs synaptic pruning (Ferro et al., 2018)	TNFR/IKK/NF-κB (Samidurai et al., 2018); Trk/MAPK (O'Neill et al., 2016); PI3K and PKA (Wigerblad et al., 2017); TNF-α/NF-κB (Xu et al., 2017; Ferro et al., 2018); TNF-α/p38 MAPK (Kleidonas et al., 2023)	Anxiety-like behaviors (Cavanagh et al., 2016); nociceptive behaviors (Wigerblad et al., 2017; Liu et al., 2017); impaired learning and memory (Wang et al., 2019; Figueiredo et al., 2019; Garre et al., 2017)
Aberrant CD200/CD200R signaling	Inhibits LTP (Ngwa and Liu, 2019); increases ATP levels (Ren et al., 2016); causes dendritic spine loss and morphological abnormalities (Sun et al., 2020); contributes to neuronal and synaptic loss (Rabaneda-Lombarte et al., 2021; Wang et al., 2020; Loh et al., 2016; Feng et al., 2019)	MyD88/TAK1/NF-κB (Jiang et al., 2016); CD200R/Foxp3 (Yi et al., 2016); p38-MAPK/JNK (Sun et al., 2020)	Depression-like behaviors (Wang et al., 2017); nociceptive behaviors (Hernangomez et al., 2016); impaired sensorimotor function (Sun et al., 2020); impaired learning and memory (Feng et al., 2019)
Aberrant CX3CL1/CX3CR1 signaling	Promotes LTP (Poniatowski et al., 2017); enhances excitatory transmission (Basilico et al., 2021); impairs dendritic spine density and morphology (Basilico et al., 2021; Bolós et al., 2018; Gellner et al., 2023); leads to synapse loss (Wang et al., 2023; Li et al., 2020); reduces axonal terminal area (Bolós et al., 2018); causes defective synaptic pruning (Gunner et al., 2019; Reshef et al., 2017)	PI3K/AKT and Wnt3a-bcatenin (Li et al., 2020); CX3CR1/CX3CL1/ADAM10 (Gunner et al., 2019)	Anxiety-like behaviors (Bolós et al., 2018); impaired learning and memory (Wang et al., 2023; Li et al., 2020; Gellner et al., 2023)
Aberrant CSF1/CSF1R signaling	Promotes LTP (Zhou et al., 2019); induces excitatory (Kwon et al., 2017; Boakye et al., 2019) and inhibitory transmission (Boakye et al., 2019); reduces dendritic spine density (Wohleb et al., 2018; Horchar and Wohleb, 2019; Bollinger et al., 2020); causes neuronal/synapse loss (Gushchina et al., 2018; Olmos-Alonso et al., 2016; Kodali et al., 2024); impairs synaptic elimination/pruning (Mein et al., 2023; Wohleb et al., 2018; Horchar and Wohleb, 2019; Bollinger et al., 2020; Chung et al., 2023; Ding et al., 2021)	CSF1R/DAP12 (Guan et al., 2016); ERK1/2 and Akt/GSK3β (Kwon et al., 2017); mTOR (Kodali et al., 2024)	Depression-like behaviors (Wohleb et al., 2018; Horchar and Wohleb, 2019; Bollinger et al., 2020; Kwon et al., 2017); anxiety-like behaviors (Wohleb et al., 2018); nociceptive behaviors (Zhou et al., 2019; Guan et al., 2016; Okubo et al., 2016); hyperactivity (Olmos-Alonso et al., 2016); impaired learning and memory (Mein et al., 2023; Horchar and Wohleb, 2019; Bollinger et al., 2020; Chung et al., 2023; Olmos-Alonso et al., 2016)
Aberrant IFN-γ/IFN-γR	Enhances excitatory transmission (Reischer et al., 2020); alters neurotransmitter release (Monteiro et al., 2016; Gallego-Delgado et al., 2020; French et al., 2019); reduces neuronal density and morphology (Monteiro et al., 2016; Tong et al., 2023); shortens dendritic length (Monteiro et al., 2016); impairs synaptic pruning (Huang et al., 2021; He et al., 2024; Garber et al., 2019; Kann et al., 2022; French et al., 2019)	LRRK2/IFN-γ (Panagiotakopoulou et al., 2020); JAK/STAT1 (He et al., 2024)	Impaired learning and memory (Monteiro et al., 2016; He et al., 2024; Garber et al., 2019)

In this context, selectively modulating pro-inflammatory cytokines and receptors may offer a more refined strategy to restore synaptic integrity and reduce disease burden. Accomplishing this goal requires a comprehensive understanding of the molecular mechanisms underpinning microglia-driven synaptic remodeling. Future investigations should focus on: (i) elucidating the intracellular and intercellular signaling pathways linked to microglial cytokines and receptors, (ii) identifying optimal cytokine/receptor targets for therapeutic intervention, and (iii) exploring how microglia-astrocyte interactions contribute to synaptic remodeling in both physiological and pathological states.
